# Pivotal Role of Fatty Acid Synthase in c-MYC Driven Hepatocarcinogenesis

**DOI:** 10.3390/ijms21228467

**Published:** 2020-11-11

**Authors:** Jiaoyuan Jia, Li Che, Antonio Cigliano, Xue Wang, Graziella Peitta, Junyan Tao, Sheng Zhong, Silvia Ribback, Matthias Evert, Xin Chen, Diego F. Calvisi

**Affiliations:** 1 Department of Bioengineering and Therapeutic Sciences and Liver Center, University of California, San Francisco, CA 94143, USA; jiajy01@163.com (J.J.); cheli0315@yahoo.com (L.C.); junyantao2010@gmail.com (J.T.); Sheng.Zhong@ucsf.edu (S.Z.); 2 Department of Oncology and Hematology, the Second Hospital, Jilin University, Changchun 130041, China; 3 Legend Biotech USA R&D Center, Piscataway, NJ 08854, USA; 4 Institute of Pathology, University of Regensburg, 93053 Regensburg, Germany; a.cyglius@gmail.com (A.C.); graziella.85@live.it (G.P.); matthias.evert@klinik.uni-regensburg.de (M.E.); 5 Department of Medical, Surgical and Experimental Sciences, University of Sassari, 07100 Sassari, Italy; 6 Department of Nutritional Sciences and Toxicology, University of California Berkeley, Berkeley, CA 94720, USA; xue.wang@berkeley.edu; 7 Institute of Pathology, University of Greifswald, 17475 Greifswald, Germany; silvia.ribback@uni-greifswald.de

**Keywords:** FASN, lipogenesis, hepatocellular carcinoma, c-Myc, mouse models

## Abstract

Hepatocellular carcinoma (HCC) is a deadly form of liver malignancy with limited treatment options. Amplification and/or overexpression of *c-MYC* is one of the most frequent genetic events in human HCC. The mammalian target of Rapamycin Complex 1 (mTORC1) is a major functional axis regulating various aspects of cellular growth and metabolism. Recently, we demonstrated that mTORC1 is necessary for c-Myc driven hepatocarcinogenesis as well as for HCC cell growth in vitro. Among the pivotal downstream effectors of mTORC1, upregulation of Fatty Acid Synthase (FASN) and its mediated de novo lipogenesis is a hallmark of human HCC. Here, we investigated the importance of FASN on c-Myc-dependent hepatocarcinogenesis using in vitro and in vivo approaches. In mouse and human HCC cells, we found that FASN suppression by either gene silencing or soluble inhibitors more effectively suppressed proliferation and induced apoptosis in the presence of high c-MYC expression. In c-Myc/Myeloid cell leukemia 1 (MCL1) mouse liver tumor lesions, FASN expression was markedly upregulated. Most importantly, genetic ablation of *Fasn* profoundly delayed (without abolishing) c-Myc/MCL1 induced HCC formation. Liver tumors developing in c-Myc/MCL1 mice depleted of *Fasn* showed a reduction in proliferation and an increase in apoptosis when compared with corresponding lesions from c-Myc/MCL1 mice with an intact *Fasn* gene. In human HCC samples, a significant correlation between the levels of c-MYC transcriptional activity and the expression of *FASN* mRNA was detected. Altogether, our study indicates that FASN is an important effector downstream of mTORC1 in c-MYC induced HCC. Targeting FASN may be helpful for the treatment of human HCC, at least in the tumor subset displaying c-MYC amplification or activation.

## 1. Introduction

Hepatocellular carcinoma (HCC) is the most frequent primary malignancy of the liver and the fourth most common cause of cancer-related death worldwide [1]. The major risk factors associated with HCC development are chronic infections by hepatitis B and C, obesity-related non-alcoholic fatty liver disease (NFALD)/non-alcoholic steatohepatitis (NASH), diabetes, and alcohol abuse [2,3]. Patients with progressed HCC have limited treatment options, most often resulting in poor outcome. The drugs available for the treatment of advanced HCC include the multi-kinase inhibitors Sorafenib, Lenvatinib, and Regorafenib, but they are generally characterized by limited efficacy [3,4,5,6,7]. Many additional drugs, including tyrosine kinase inhibitors and Rapalogs, have been tested in clinical trials, but none of them showed superior efficacy when compared to Sorafenib [3,8,9]. Thus, there is an obvious unmet medical need for new drugs for the treatment of HCC.

c-MYC, a well-characterized proto-oncogene in many organs including the liver, is a transcription factor that induces cellular transformation and tumor progression. The critical role of c-MYC in hepatocarcinogenesis has been proven undoubtedly by the finding that c-MYC overexpression suffices to trigger HCC development in mice [10,11,12]. It has been well established that c-MYC is a potent inducer of apoptosis [13,14,15]. This is a part of the normal defense mechanisms to eliminate cells which have potential to become cancer cells. Therefore, preventing c-MYC induced program cell death is one of the key factors during c-MYC driven tumorigenesis [13,14,15]. Indeed, upregulation or aberrant activation of anti-apoptosis genes are frequently observed in tumors. One of the key anti-apoptotic genes involved in c-MYC driven cancer development is Myeloid cell leukemia 1 (MCL1). MCL1, a member of the B-cell lymphoma 2 (BCL2) protein family, localizes to the outer mitochondrial membrane, as well as in other intracellular membranes. Overexpression of MCL1 has been shown to potentiate c-MYC induced lymphoma development via suppressing c-MYC driven apoptosis [16]. Similar results were observed in non-small cell lung cancer [17]. MCL1 and c-MYC also cooperate to promote chemo-resistance in breast cancer [18]. MCL1 overexpression has been reported in HCC, where it associates with low tumor differentiation grade and poor patients’ prognosis [19,20]. Similar to c-MYC, MCL1 has been found to be necessary for the development of HCC in mice and humans [20,21]. Importantly, studies have found that while overexpression of c-MYC alone is unable to drive HCC formation in mice of C57BL/6 background due to extensive c-MYC induced hepatocyte death [22], concomitant overexpression of c-MYC and MCL1 promoted hepatocarcinogenesis in this genetic background [23]. 

Fatty Acid Synthase (FASN) plays a key role in lipid biosynthesis. FASN is strongly expressed in the developing embryo as well as in selected adult organs, such as liver, brain, adipose tissue, endometrium, seminal vesicles, and mammary gland, where lipogenesis is a critical process. In striking contrast, low levels of FASN characterize other rapidly dividing normal tissues, such as the gastric and intestinal epithelium, and the hematopoietic compartment [24,25]. In addition, increased levels of FASN and related de novo lipogenesis have been observed in multiple cancer types, including tumors of the breast, gastrointestinal tract, prostate, bladder, ovary, lung, oral cavity, and head and neck [26,27]. In human HCC, previous studies conducted by several groups including ours revealed the augmented and coordinated expression of the major enzymes responsible for de novo lipogenesis, including FASN, ATP citrate lyase (ACLY), acetyl-CoA carboxylase (ACC), and stearoyl-coA desaturase (SCD) [28,29]. Importantly, the increased levels of pro-lipogenic proteins were found to be inversely correlated with the length of patients’ survival in this aggressive disease [29]. Mechanistically, we discovered that the increased de novo lipogenesis is controlled by the AKT/mTORC1/S6/SREBP1 signaling pathway in HCC [29]. Subsequent experiments revealed that silencing of FASN decreases HCC cell growth and increases apoptosis in vitro, and ablation of *FASN* completely suppresses AKT and AKT/c-Met driven HCC formation in mice [25,30]. Altogether, these studies provide the evidence, for the first time, that FASN and its mediated lipogenesis are required for HCC growth in vivo [29]. Intriguingly, in a successive study, we found that genetic deletion of *FASN* does not affect hepatocarcinogenesis driven by co-expression of *β-catenin* and *c-Met* protooncogenes in the mouse liver [28]. The latter finding suggests that the contribution of FASN-driven lipogenesis is either required or dispensable for liver tumorigenesis, depending on the oncogenes involved [28]. Consequently, therapeutic inhibition of FASN activity might be either highly detrimental or ineffective for HCC treatment in molecularly different tumor subsets. Recently, we found that an intact mTORC1 axis is needed for c-Myc-driven hepatocarcinogenesis [31]. Furthermore, it has been revealed that c-MYC cooperates with SREBP1 to induce lipogenesis and promote neoplastic liver growth [32]. However, the specific contribution of FASN along hepatocarcinogenesis induced by the c-MYC oncoprotein has never been investigated to date.

In the present study, we determined the functional relevance and the possible therapeutic role of FASN on c-MYC dependent hepatocarcinogenesis by employing in vitro and in vivo approaches.

## 2. Results

### 2.1. FASN Inactivation Is Detrimental for the Growth of c-MYC HCC Cell Lines

**Figure 1:**
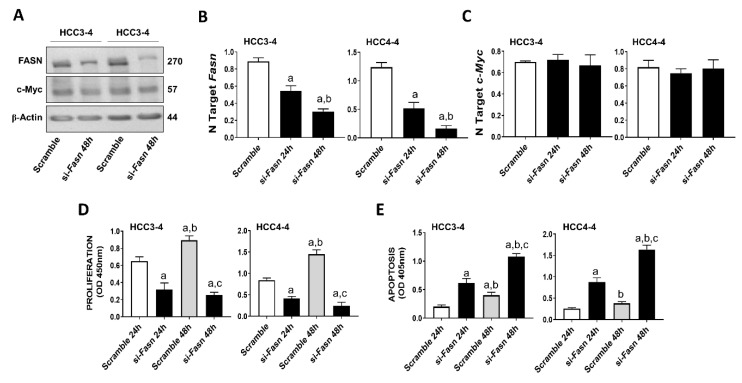
Suppression of FASN is highly detrimental for the growth of c-Myc HCC3-4 and HCC4-4 derived mouse HCC cell lines. (**A**) Western blot analysis of HCC3-4 and HCC4-4 cells subjected to scramble or FASN-specific siRNA (si-*Fasn*). (**B**) Quantitative real-time RT-PCR showing effective downregulation of *Fasn* following siRNA-mediated siRNA. (**C**) Quantitative real-time RT-PCR showing that FASN knockdown does not affect *c-Myc* mRNA levels in the two cell lines. (**D**) Effect of *Fasn* silencing on the proliferation rate of HCC3-4 and HCC4-4 cell lines. (**E**) Effect of *Fasn* silencing on the apoptosis degree of HCC3-4 and HCC4-4 cell lines. Tukey–Kramer test: at least *p* < 0.01; a, vs. scramble or scramble 24 h; b, vs. si-*Fasn* 24 h; c, vs. scramble 48 h.

Recently, it has been demonstrated that c-MYC induced growth is severely hindered by the inhibition of the mTORC1/SREBP1 pathway [32]. Here, we evaluated the specific contribution of FASN in this process, namely we assessed whether FASN suppression affects the growth of c-MYC liver cancer cells in vitro. For this purpose, the HCC3-4 and HCC4-4 mouse HCC cell lines, which were previously derived from c-Myc transgenic mice and display a robust expression of c-Myc [33], were subjected to *Fasn* inhibition by specific small interfering RNA (siRNA; Figure 1). We found that *Fasn* silencing resulted in the decrease of FASN protein and mRNA levels (Figure 1A,B), whereas the c-Myc corresponding levels were unaffected by *Fasn* gene knockdown (Figure 1A,C). Of note, *Fasn* silencing effectively suppressed the growth of HCC3-4 and HCC4-4 cell lines by decreasing proliferation (Figure 1D) and inducing apoptosis (Figure 1E). 

**Figure 2:**
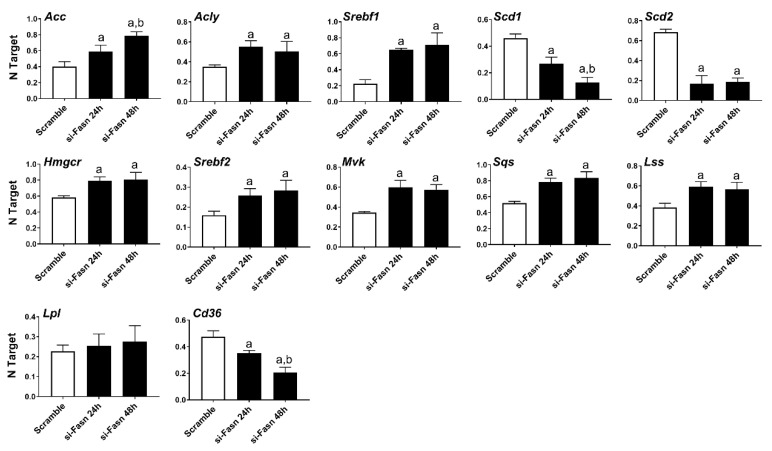
Effects of suppression of FASN by Fasn-specific siRNA (si-*Fasn*) on the levels of major lipogenic genes in the HCC3-4, c-Myc derived, mouse HCC cell line, as assessed by quantitative real-time RT-PCR. Experiments were conducted three times in triplicate. Tukey–Kramer test: at least *p* < 0.01; a, vs. Scramble; b, vs. si-*Fasn* 24 h.

Subsequently, we evaluated the effect of *Fasn* silencing on the levels of major lipogenic genes in the HCC3-4 (Figure 2) and HCC4-4 (Figure S1) cell lines. In the fatty acid synthesis pathway, knockdown of *Fasn* was accompanied by the slight upregulation of the upstream inducers of Fasn (*Acly*, *Acc, Srebf1*), whereas the downstream effectors of Fasn (*Scd1* and *Scd2*) were downregulated. Cholesterol biosynthesis genes (*Hmgcr*, *Srepf2*, *Mvk*, *Sqs*, and *Lss*) were all significantly but marginally upregulated (<2-folds) following *Fasn* depletion. No significant differences in the levels of the fatty acid transporter lipoprotein lipase or *Lpl* were detected, while *Cd36* was downregulated when comparing *Fasn*-silenced cell lines to corresponding controls. 

**Figure 3:**
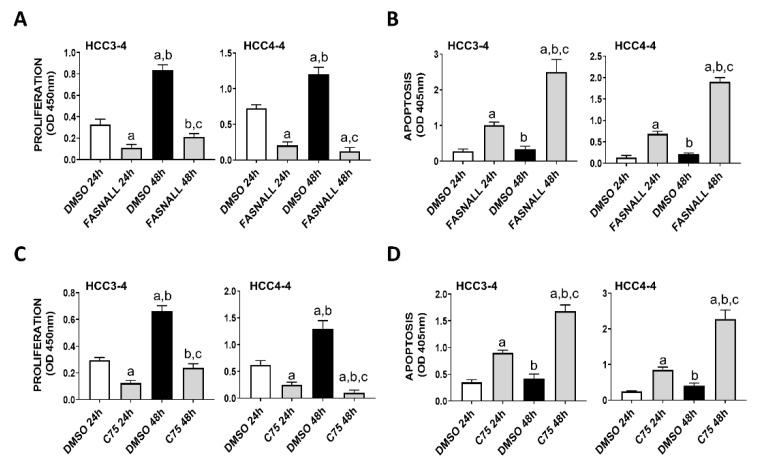
Suppression of FASN by specific inhibitors is highly detrimental for the growth of c-Myc HCC3-4 and HCC4-4 derived mouse HCC cell lines. (**A**,**B**) Effect of the FASN inhibitor FASNALL (20 µM) on the proliferation rate (**A**) and apoptosis (**B**) of HCC3-4 and HCC4-4 cell lines. (**C**,**D**) Effect of the FASN inhibitor C75 (50 µM) on the proliferation rate (**C**) and apoptosis (**D**) of HCC3-4 and HCC4-4 cell lines. Tukey–Kramer test: at least *p* < 0.01; a, vs. DMSO 24 h (drug solvent); b, vs. FASNALL or C75 24 h; c, vs. DMSO 48 h.

Next, we determined whether similar effects on c-Myc associated growth could be achieved by the inhibition of FASN activity on the same cell lines. Noticeably, strong decline of proliferation and induction of apoptosis were detected in the HCC3-4 and HCC4-4 mouse HCC cell lines following the administration of the FASN inhibitors FASNALL and C75 (Figure 3). 

**Figure 4:**
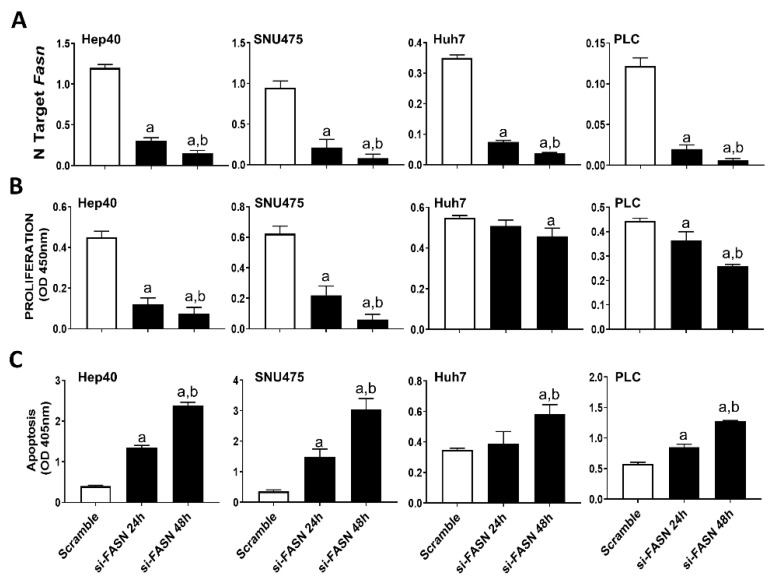
Suppression of FASN is highly detrimental for the growth of human HCC cell lines displaying high levels of c-MYC. (**A**) Quantitative real-time RT-PCR showing effective downregulation of FASN following siRNA-mediated siRNA in the Hep40, SNU475, Huh7, and PLC cell lines. (**B**) Effect of *FASN* silencing on the proliferation rate of Hep40, SNU475, Huh7, and PLC cell lines. (**C**) Effect of *FASN* silencing on the apoptosis of Hep40, SNU475, Huh7, and PLC cell lines. Tukey–Kramer test: at least *p* < 0.01; a, vs. scramble; b, vs. si-*FASN* 24 h.

**Figure 5:**
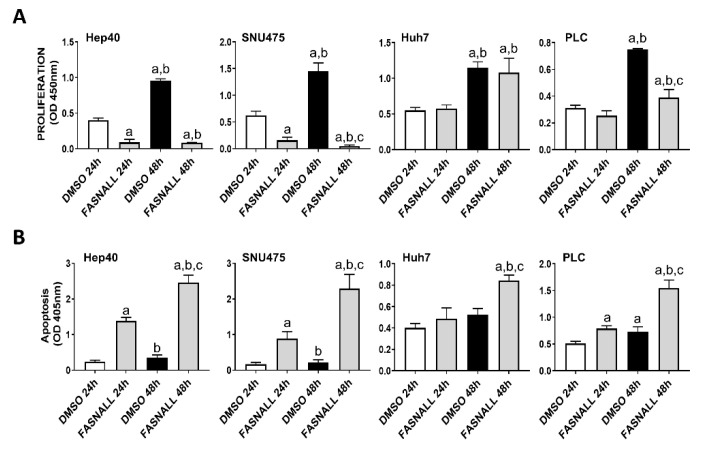
Suppression of FASN by the FASNALL specific inhibitor is highly detrimental for the growth of human HCC cell lines displaying high levels of c-MYC. (**A**) Effect of the FASN inhibitor FASNALL (20 µM) on the proliferation rate of c-MYC-high (Hep40 and SNU475) and c-MYC-low (PLC and Huh7) cell lines. (**B**) Effect of the FASN inhibitor FASNALL (20 µM) on the apoptosis of c-MYC-high (Hep40 and SNU475) and c-MYC-low (PLC and Huh7) cell lines. Tukey–Kramer test: at least *p* < 0.01; a, vs. DMSO 24 h (drug solvent); b, vs. FASNALL 24h; c, vs. DMSO 48 h.

Our previous study assessed the c-MYC expression in a collection of human hepatoma cell lines, and found that Hep40, SNU475, and SK-Hep1 cells display the highest levels of c-MYC, whereas PLC, Hep3B, and Huh7 cells exhibited the lowest c-MYC expression [31]. Similar to that observed in mouse cell lines, despite a similar suppression of *FASN* levels by specific siRNA (Figure 4A), a more pronounced growth restraint (i.e., higher decline of proliferation and stronger induction of apoptosis) was achieved in Hep40 and SNU475 cell lines (exhibiting high c-MYC expression) than in Huh7 and PLC cells (expressing low c-MYC levels). Although the lowest c-MYC levels were detected in PLC cells, the smallest growth inhibition was achieved in Huh7 cells by FASN silencing (Figure 4B,C). A similar pattern was detected when the same cell lines were subjected to administration of FASNALL (Figure 5).

**Figure 6:**
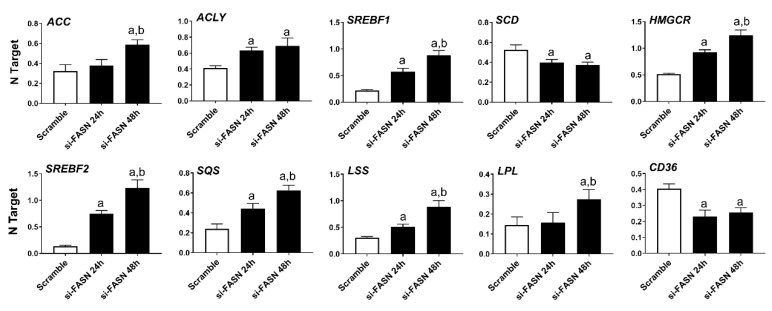
Effects of suppression of FASN by FASN-specific siRNA (si-*FASN*) on the levels of major lipogenic genes in the Hep40 human HCC cell line, as assessed by quantitative real-time RT-PCR. Experiments were conducted three times in triplicate. Tukey–Kramer test: at least *p* < 0.01; a, vs. Scramble; b, vs. si-*FASN* 24 h.

As concerns the members of the fatty biosynthesis pathway, knockdown of *FASN* resulted in the induction of *ACLY*, *ACC*, and *SREBF1* genes as well as the downregulation of the *SCD* gene in all human HCC cell lines tested, with no overt differences. Cholesterol biosynthesis genes (*HMGCR*, *SREBF2*, *SQS*, and *LSS*) were all upregulated following *FASN* silencing. Furthermore, silencing of FASN induced upregulation of the fatty acid transporter *LPL* and downregulation of the fatty acid transporter *CD36* in the four human HCC cell lines (Figure 6, Figures S2–S4).

In summary, our data indicate that FASN suppression effectively inhibits c-MYC induced in vitro growth. 

### 2.2. FASN Is Not a Direct Downstream Effector of c-MYC in Mouse and Human HCC Cell Lines

**Figure 7:**
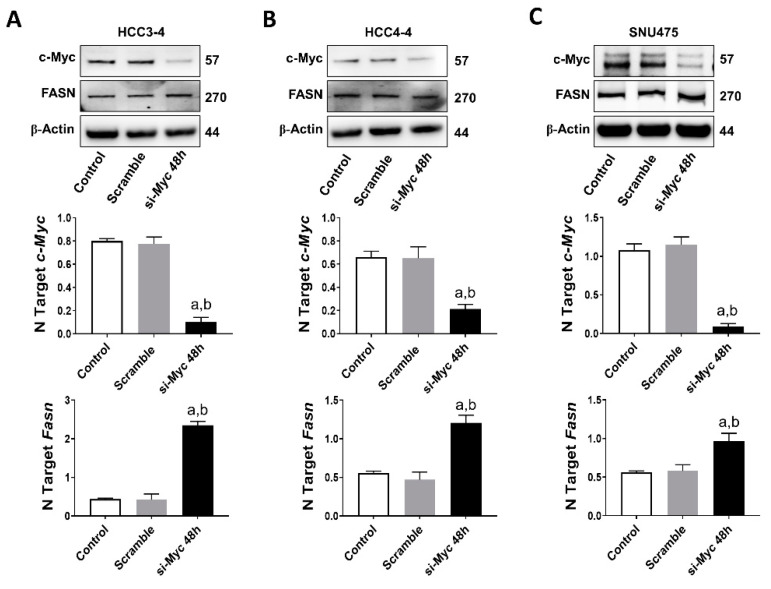
FASN is not a direct c-MYC target in mouse and human HCC cell lines. (**A**) Effect of c-Myc silencing on the protein (upper panel) and mRNA levels of *c-Myc* (middle panel) and *Fasn* (lower panel) in the HCC3-4 mouse HCC cell line. (**B**) Effect of c-Myc silencing on the protein (upper panel) and mRNA levels of *c-Myc* (middle panel) and *Fasn* (lower panel) in the HCC4-4 mouse HCC cell line. (**C**) Effect of c-MYC silencing on the protein (upper panel) and mRNA levels of *c-MYC* (middle panel) and *FASN* (lower panel) in the SNU475 human HCC cell line. Tukey–Kramer test: at least *p* < 0.01; a, vs. control (untreated); b, vs. Scramble (solvent).

Next, based on the assumption that c-MYC is a major transcriptional activator in the genome [34], we investigated the possibility that FASN could also be a direct downstream target of c-MYC in HCC. For this purpose, we performed an in silico prediction of potential binding sites for c-MYC on the mouse and human *FASN* gene promoter using the EPDnew software [35]. Noticeably, we identified several putative binding sites for c-MYC on the promoter region of both human and mouse *FASN* (*p* < 0.001; Figure S5A,B), situated within -950 and -50 base pairs from the ATG starting codon. Based on these encouraging findings, *c-Myc* was knocked down in mouse HCC3-4 and HCC4-4 cell lines as well as in the human SNU475 cells by siRNA. We found that siRNA-mediated silencing of *c-Myc* did not affect protein levels of FASN in the three cell lines. Furthermore, a paradoxical upregulation of *FASN* mRNA was detected in the three cell lines following *c-Myc* knockdown (Figure 7A–C). On the other hand, suppression of mTORC1 via the partial mTORC1 inhibitor Rapamycin (Figure S6A) or by specific siRNA-mediated silencing of the mTORC1 component *Raptor* or the mTORC1 downstream effector ribosomal protein S6 (S6; Figure S6B) led to a remarkable downregulation of FASN in HCC3-4 and HCC4-4 cell lines. Equivalent results were obtained when applying the same approaches to Hep40 and SNU475 human HCC cell lines (Figure S7A,B), implying mTORC1 as the major regulator of FASN levels in mouse and human HCC cell lines with elevated c-MYC expression. Of note, our data are in accordance with the recent findings by Gouw et al. [24], indicating that c-MYC alone is unable to drive the transcriptional activation of *FASN* (and other lipogenic genes), but requires the functional cooperation with SREBP1/SREBF1 (a main mTORC1 target) to achieve this goal. 

Overall, the present findings indicate that FASN is not directly regulated by c-MYC in mouse and human HCC cells. 

### 2.3. Ablation of Fasn Significantly Delays c-Myc/MCL1 Induced Hepatocarcinogenesis in Mice

**Figure 8:**
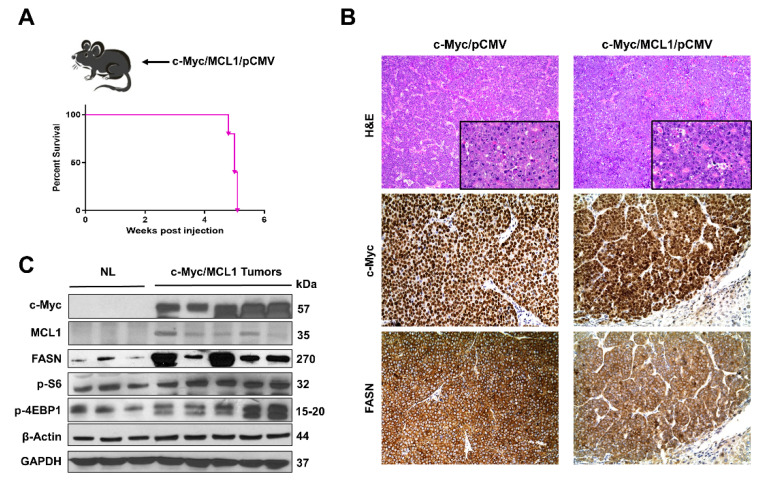
Development of liver tumors in C57BL/6 mice subjected to hydrodynamic tail vein gene delivery of the c-Myc and MCL1 plasmids. (**A**) Scheme of the injection strategy and survival curve of c-Myc/MCL1 mice. (**B**) Histopathological features of liver lesions developed in c-Myc (c-Myc/pCMV; FVB/N genetic background) and c-Myc/MCL1 (c-Myc/MCL1/pCMV; C57BL/6 genetic background) mice. Note in both models the development of hepatocellular tumors, consisting of small, highly basophilic cells (better appreciable in insets), as shown by hematoxylin and eosin (H&E) staining. Both c-Myc and c-Myc/MCL1 mice exhibit strong immunoreactivity for c-Myc and FASN proteins. Original magnifications: 100× in H&E, 400× in insets, c-Myc, and FASN immunohistochemistry. (**C**) Representative Western blot analysis showing the induction of hydrodynamically transfected proteins (c-Myc and MCL1) as well as of FASN and targets of the mTORC1 complex (p-S6 and p-4EBP1) in the tumor lesions of c-Myc/MCL1 mice. GAPDH and β-Actin antibodies were used as loading controls.

Subsequently, we determined whether the in vitro findings could be translated in vivo. Previous data from our group indicate that hydrodynamic gene delivery of the c-Myc oncogene into the liver of C57BL/6 mice does not lead to HCC development, a phenomenon that is successfully circumvented by co-expression of the MCL1 protooncogene [23]. Specifically, the co-expression of c-Myc and MCL1 in the C57BL/6 mouse strain leads to the development of HCC that are morphologically and moleculary undistinguishable from tumors occurring in FVB/N mice overexpressing c-Myc alone [23]. As a consequence, the c-Myc/MCL1 mouse is accepted as a proper c-Myc model for HCC in C57BL/6 mice [23]. We found that forced overexpression of c-Myc and MCL1 protooncogenes in C57BL/6 mice (referred to as c-Myc/MCL1 mice) by hydrodynamic injection triggered rapid liver tumorigenesis. Indeed, by five weeks after injection, 100% of c-Myc/MCL1 mice developed high liver tumor burden with palpable abdominal masses and were euthanized based on our Institutional Animal Care and Use Committee protocol (Figure 8A). Histologically, hepatocellular tumors from c-Myc/MCL1 mice consisted of small, highly basophilic cells, thus being indistinguishable from tumor lesions developed in c-Myc mice in the FVB/N genetic background (Figure 8B), in accordance with previous findings [23]. By immunohistochemistry, c-Myc/MCL1 tumors exhibited strong immunoreactivity for c-Myc and FASN proteins (Figure 8B). As expected, c-Myc/MCL1 mouse tumors showed higher levels of c-Myc and MCL1 proteins than wild-type, uninjected livers, as assessed by Western blot analysis. Furthermore, c-Myc/MCL1 lesions displayed increased amount of mTORC1 effectors, such as FASN, phosphorylated/activated S6 (p-S6) and inactivated/phosphorylated 4EBP1 (p-4EBP1) than wild-type livers (Figure 8C), equivalent to that occurring in c-Myc-only overexpressing liver lesions [31].

**Figure 9:**
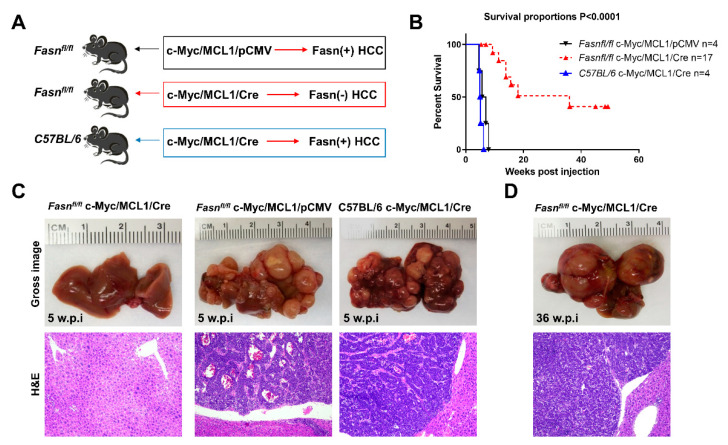
Genetic ablation of FASN strongly delays c-Myc/MCL1 hepatocarcinogenesis in mice. (**A**) Scheme of the hydrodynamic gene delivery strategy. In the control group (*Fasn^fl/fl^* injected with c-Myc/MCL1 genes; upper panel), the tumors display immunoreactivity for FASN (Fasn + HCC). In the conditional knockout group (*Fasn^fl/fl^* injected with c-Myc/MCL1 and Cre genes; middle panel), the tumors show overexpression of c-Myc/MCL1 protooncogenes but are Fasn negative (Fasn − HCC). To exclude off-targets effects of the Cre recombinase, C57BL/6 mice were also injected with c-Myc/MCL1 and Cre genes; lower panel). (**B**) Survival curves of the three groups of mice. (**C**) Histopathological features of c-Myc/MCL1/Cre, c-Myc/MCL1, and C57BL/6/c-Myc/MCL1 livers 5 weeks post hydrodynamic injection. Macroscopically, livers from c-Myc/MCL1 and C57BL/6/c-Myc/MCL1 mice were occupied by large nodular lesions, whereas c-Myc/MCL1/Cre livers appeared normal. Microscopically, large tumors composed of highly basophilic cells occupied most of the c-Myc/MCL1 and C57BL/6/c-Myc/MCL1 liver parenchyma, while no appreciable lesions could be observed in c-Myc/MCL1/Cre mice. (**D**) After a long latency, 36 weeks post injection, c-Myc/MCL1/Cre mice also developed tumors. Original magnifications: 100× in hematoxylin and eosin stained slides.

To investigate the requirement of FASN and mediated de novo lipogenesis in c-Myc/MCL1 driven hepatocarcinogenesis, conditional *Fasn* KO mice (*Fasn^fl/fl^* mice) [25,36] were applied. Thus, we hydrodynamically injected c-Myc, MCL1, and Cre plasmids into *Fasn^fl/fl^* mice (that will be referred to as c-Myc/MCL1/Cre mice; *n* = 17), thus allowing the overexpression of c-Myc and MCL1 oncogenes and deletion of *Fasn* in the same subset of mouse hepatocytes. As a control, c-Myc, MCL1, and pCMV (empty vector) constructs were co-injected into *Fasn^fl/fl^* mice (c-Myc/MCL1/pCMV; *n* = 4) (Figure 9A). As an additional control, to exclude unspecific effects by the Cre recombinase, wild-type C57BL/6 mice were injected with c-Myc, MCL1, and Cre plasmids (C57BL/6/c-Myc/MCL1/Cre mice; Figure 9B). While all c-Myc/MCL1/pCMV mice and C57BL/6/c-Myc/MCL1/Cre mice developed high liver tumor burden by 5–6 weeks post-injection (Figure 9C), livers of c-Myc/MCL1/Cre mice appeared to be normal at the same time point (Figure 9C). Nonetheless, after a long latency, also c-Myc/MCL1/Cre developed neoplastic lesions and reached an elevated tumor burden by 36 weeks post injection (Figure 9D). Tumors were histopathologically identical to those developed in c-Myc/MCL1 mice, implying that the absence of FASN does not modify the tumor phenotype in this mouse model (Figure 9C,D). Indeed, liver tumors from c-Myc/MCL1/pCMV and c-Myc/MCL1/Cre mice were composed of small, round, and highly basophilic cells with hyperchromatic nuclei, growing either as a solid pattern, arranged in thick chords of cells demarcated by a thin layer of fibrovascular stroma (macrotrabecular pattern), or forming acinar/pseudoglandular structures. These three growth patterns most often coexisted in liver tumors from c-Myc/MCL1/pCMV and c-Myc/MCL1/Cre mice (Figure S8). 

Altogether, the present results indicate that *Fasn* activation is an important molecular event in c-Myc/MCL1 induced hepatocarcinogenesis. However, oncogene-expressing hepatocytes could eventually overcome the loss of *Fasn* and progress into HCC after a long latency.

### 2.4. Analysis of Fasn Regulated Metabolic and Growth-Related Pathways along c-Myc/MCL1 Hepatocarcinogenesis

**Figure 10:**
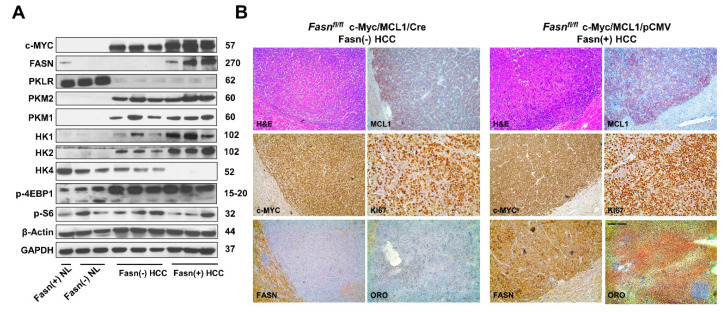
Metabolic and molecular features of c-Myc/MCL1/Cre and c-Myc/MCL1/pCMV mice as assessed by Western blot analysis (**A**) and immunohistochemistry (**B**). Abbreviation: H&E, hematoxylin and eosin staining. Original magnifications: 100× in H&E, MCL1, c-Myc, FASN, and ORO staining; 200× in Ki67 stained slides.

Subsequently, we compared the molecular features of c-Myc/MCL1/Cre and c-Myc/MCL1/pCMV mouse tumor lesions (Figure 10). Western blot analysis revealed that HCC lesions from both c-Myc/MCL1/Cre and c-Myc/MCL1/pCMV cohorts expressed the transfected c-Myc gene. As expected, FASN expression was lost in c-Myc/MCL1/Cre HCCs, confirming *Fasn* effective deletion by the Cre recombinase system (Figure 10A). Furthermore, c-Myc/MCL1/Cre mice displayed lower levels of glycolysis proteins hexokinase (HK) HK1 and HK2 when compared with that observed in c-Myc/MCL1/pCMV mice. While levels of p-S6, p-4EBP1 and pyruvate kinase isozymes (PKM1/2) were equivalent in c-Myc/MCL1/Cre and c-Myc/MCL1/pCMV liver tumors, levels of HK4 were undetectable in c-Myc/MCL1/pCMV lesions. In addition, levels of Pyruvate Kinase L/R (PKLR) were very low in both c-Myc/MCL1/Cre and c-Myc/MCL1/pCMV mice when compared with normal livers from uninjected mice (Figure 10A). At the immunohistochemical level, c-Myc/MCL1/Cre and c-Myc/MCL1/pCMV tumor lesions displayed robust immunoreactivity for c-Myc and MCL1 with no overt differences (Figure 10B). Effective suppression of *Fasn* and related lipogenesis was confirmed by the absence of FASN immunolabeling and the lipid Oil-Red-O staining, whereas strong immunoreactivity was observed for the same parameters in c-Myc/MCL1/pCMV tumors. Strong immunolabeling for the proliferation marker Ki67 was detected in the two mouse models, with an apparently higher number of Ki67 positive cells in c-Myc/MCL1/pCMV lesions (Figure 10B).

**Figure 11:**
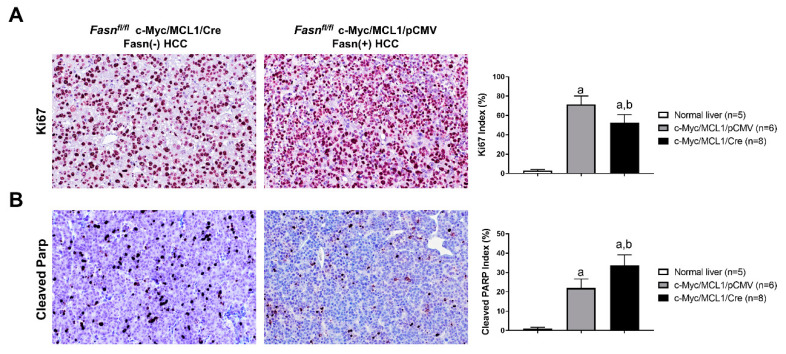
Comparison of proliferation and apoptosis rates in liver lesions from c-Myc/MCL1/pCMV and c-Myc/MCL1/Cre mice. (**A**) Graphs and representative tissue images of proliferation rates in the two mouse models as assessed by evaluating the immunolabeling for Ki67. (**B**) Graphs and representative tissue images of apoptotic rates in the two mouse models as assessed by evaluating the percentage of cells positive for the Cleaved-Parp antibody. Data are expressed as means ± SD. A total of five normal livers, 6 liver tumor lesions from c-Myc/MCL1/pCMV, and 8 liver tumor lesions from c-Myc/MCL1/Cre mice were analyzed. Original magnification: 200×. Tukey–Kramer test: at least *p* < 0.0003; a, vs. normal liver; b, vs. c-Myc/MCL1/pCMV mice.

Following the impression that c-Myc/MCL1/pCMV mouse lesions display a higher immunoreactivity for the proliferation marker Ki67, we assessed the Ki67 index in tumors from c-Myc/MCL1/Cre (*n* = 8) and c-Myc/MCL1/pCMV mice (*n* = 6, consisting of the four mice from the present investigation and two additional mice from a previous study [28]). As expected, while normal livers showed a low rate of proliferation, as assessed by the percentage of Ki67 positive cells (*n* = 5; 2.8 ± 1.1), both models exhibited a robust proliferation in the tumor lesions, with c-Myc/MCL1/pCMV tumors displaying a significantly higher Ki67 index than c-Myc/MCL1/Cre corresponding lesions (53.8 ± 3.5 vs. 67.3 ± 5.8; *p* < 0.0003; Figure 11A). In contrast, a more pronounced apoptosis, as assessed by cleaved Parp staining, was detected in c-Myc/MCL1/Cre than in c-Myc/MCL1/pCMV tumors (33.11 ± 5.10 vs. 23.6 ± 4.66; *p* < 0.0001; Figure 11B), whereas very low apoptosis characterized normal livers (Figure 11B). Thus, the present data indicate that, although HCCs still develop in c-Myc/MCL1/Cre mice despite the absence of Fasn, these tumors grow significantly slower than those formed in c-Myc/MCL1/pCMV mice. 

**Figure 12:**
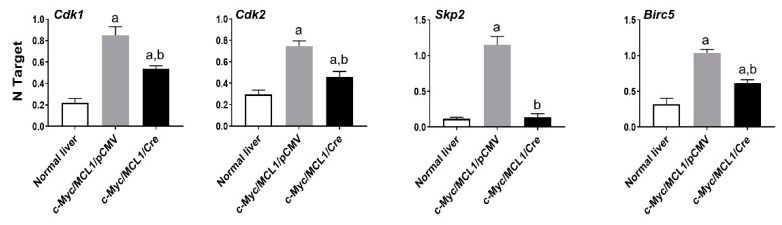
Quantitative real-time RT-PCR analysis of levels of putative FASN proliferation (*Cdk1*, *Cdk2*, *Skp2*) and anti-apoptosis (*Birc5/Survivin*) target genes in normal livers and tumor lesions of c-Myc/MCL1/pCMV and c-Myc/MCL1/Cre mice. Quantitative values for each gene were calculated by using the PE Biosystems Analysis software and expressed as number target (NT). NT = 2^−Δ*C*t^, wherein ΔCt value of each sample was calculated by subtracting the average Ct value of the target gene from the average Ct value of the *β-Actin* gene. Tukey–Kramer test: at least *p* < 0.001; a, vs. normal liver; b, vs. c-Myc/MCL1/pCMV mice.

Recently, it has been shown that the oncogenic activity of FASN might reside in its capacity to affect cell proliferation and anti-apoptotic pathways rather than in FASN pro-lipogenic function [30,31]. In the latter investigations, in particular, it has been found that FASN inhibition triggers the downregulation of various genes promoting cell proliferation and survival in cancer cells [30,31]. Inspired by our present findings showing a lower proliferation and higher apotpotic rates in tumors genetically deprived of FASN, we assessed the levels of some of the identified FASN targets [30,31] in normal wild-type livers as well as in c-Myc/MCL1/pCMV and c-Myc/MCL1/Cre mouse HCC lesions (Figure 12). Of note, we found that the levels of the cell cycle positive regulators *Cdk1*, *Cdk2*, and *Skp2* as well as those of the anti-apoptotic mediator *Birc5/Survivin* were significantly higher in c-Myc/MCL1/pCMV than in c-Myc/MCL1/Cre liver tumors. In addition, lowest levels of *Cdk1*, *Cdk2*, and *Birc5/Survivin* were detected in normal livers, whereas the expression of *Skp2* was equivalent in c-Myc/MCL1/Cre lesions and normal livers (Figure 12). 

Altogether, the present findings indicate that Fasn ablation profoundly delays but does not abolish hepatocarcinogenesis driven by co-expression of c-Myc and MCL1 protooncogenes in mice. Importantly, liver tumors developing in c-Myc/MCL1/Cre mice grow at significantly slower pace than corresponding lesions from c-Myc/MCL1/pCMV mice.

### 2.5. Suppression of Fasn Does Not Trigger Cholesterol Synthesis or Lpl/Cd36 Fatty Acid Uptake in c-Myc/MCL1 Tumors

As reported above, the present data indicate that, despite Fasn suppression, c-Myc/MCL1 lesions are able to form tumors albeit after long latency. Besides the growth properties of tumors deprived of Fasn, we next investigated the possible metabolic mechanisms compensating the loss of *Fasn* in c-Myc/MCL1 mouse tumors. Since previously we demonstrated that HCC can compensate the loss of Fasn-dependent fatty acid production by increasing cholesterogenesis [37], we assessed the levels of cholesterol synthesis genes, such as Hydroxy-Methylglutaryl-CoA Reductase (*Hmgcr*), Mevalonate Kinase (*Mvk*), Squalene Synthase (*Sqs*) and Lanosterol Synthase (*Lss*), as well as the master cholesterol regulator, Sterol Regulatory Element Binding Transcription Factor 2 (*Srebf2*), in c-Myc/MCL1/Cre and c-Myc/MCL1/pCMV livers. Significantly higher levels of *Hmgcr*, *Mvk*, *Sqs*, *Lss*, and *Srebf2* were detected in c-Myc/MCL1/pCMV livers (Figure S9), suggesting that cholesterol synthesis does not compensate for Fasn-related lipogenesis in c-Myc/MCL1 mice. Next, we analyzed the levels of the major fatty acid transporterts, namely lipoprotein lipase (LPL) and CD36. Specifically, LPL is the enzyme responsible for extracellular lipolysis of triglycerides into fatty acids, which are subsequently taken up by the hepatocytes via the CD36 receptor [38]. We found that the expression levels of *Lpl* and *Cd36* were equivalent in c-Myc/MCL1/Cre and c-Myc/MCL1/pCMV tumor samples (Figure S9), indicating the lack of a compensatory induction of the Lpl/Cd36 axis as a response to *Fasn* depletion in c-Myc mice.

Overall, the present data indicate that loss of *Fasn* does not trigger compensatory induction of cholesterogenesis or fatty acid uptake in c-Myc/MCL1 mice.

### 2.6. Levels of c-MYC Activity Correlate with FASN Expression in Human HCC

**Figure 13:**
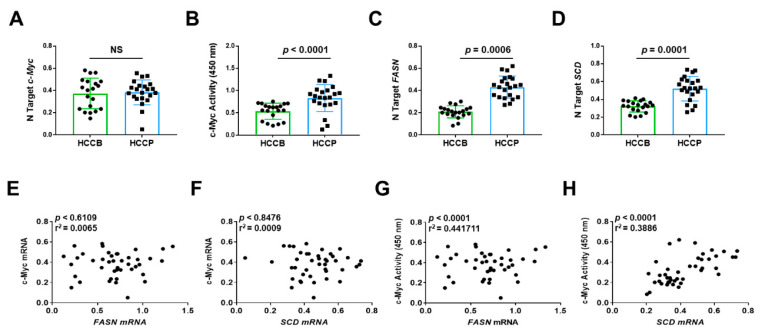
Expression levels of the *FASN* and *SCD* are directly correlated with c-MYC activity in human hepatocellular carcinoma. (**A**,**C**,**D**) Quantitative real-time RT-PCR analysis of *c-MYC*, *FASN,* and *SCD* mRNA levels in hepatocellular carcinomas with better (HCCB; *n* = 20) and poorer prognosis (HCCP; *n* = 22). Quantitative values were calculated by using the PE Biosystems Analysis software and expressed as number target (N Target). N Target = 2^−Δ*C*t^, wherein the ΔCt value of each sample was calculated by subtracting the average Ct value of the gene of interest from the average Ct value of the *β-Actin* gene. The *p*-value was calculated using two-tailed Student’s *t* test. Abbreviation: NS, not significant. (**B**) Evaluation of c-MYC activity in the same HCC collection. (**E**,**F**) Absence of significant correlation between *FASN* and *SCD* with *c-MYC* mRNA levels, as assessed by linear regression analysis. (**G**,**H**) Significant, positive correlation occurs between mRNA levels of *FASN* and *SCD* with c-MYC activity using the same statistical approach.

Finally, we determined the relationship between the expression levels of *c-MYC*, and those of *FASN* and FASN pro-lipogenic target Stearoyl-CoA Desaturase (*SCD*) in human HCC samples (*n* = 42). Because it has been established that transcriptional activity of c-MYC (and related transcriptional signature) rather than *c-MYC* mRNA levels is a reliable predictor of poor prognosis in human HCC [34], we evaluated the correlation of *FASN* mRNA levels not only with *c-MYC* mRNA expression but also with c-MYC transcriptional activity. Levels of *FASN*, *SCD*, and c-MYC activity, but not *c-MYC* mRNA, were significantly associated with poorer survival in HCC patients (Figure 13). Similarly, no significant correlation was detected between *c-MYC* mRNA levels and those of *FASN* and *SCD*, whereas a significant, positive correlation was found between *FASN* and *SCD* mRNA levels and those of c-MYC transcriptional activity (Figure 13). 

Overall, our data indicate that elevated levels of both active c-MYC and *FASN* gene are predictors of poor prognosis in this aggressive tumor, and suggest the functional interplay between c-MYC and FASN also in human HCC, as demonstrated in in vitro and in vivo models.

## 3. Discussion

Despite the significant improvement in the understanding of the molecular pathogenesis of HCC, effective therapeutic approaches for this disease in advanced stages remain an unmet need [1]. Several molecular-based therapies have been applied in preclinical and clinical trials for HCC to date, with either no or limited benefits for the patients in terms of survival [2,3,4,5,6,7,8,9]. Thus, it is likely that alternative molecular candidates should be selected and targeted to achieve a better patient outcome. 

A wealth of evidence points to the c-MYC transcription factor as a potential novel target for liver cancer treatment. Indeed, dysregulated induction of c-MYC because of its overexpression, inhibition of ubiquitination, translocation, and/or amplification is recognized as one of the main oncogenic events in rodent and human HCC [10,11,12]. However, c-MYC represents the prototypical example of an “undruggable” target, and its direct inhibition has been found to be extremely problematic to achieve [39]. Thus, strategies aiming at identifying and targeting c-MYC interactors or downstream effectors, whose inhibition is able to blunt c-MYC pro-tumorigenic activity, are of prime importance to circumvent these therapeutic limitations. Previous data from our laboratory indicate that c-MYC requires an intact mTORC1 signaling pathway to exerts its oncogenic properties and that, for this scope, c-MYC transcriptionally activates multiple amino acid transporters, ultimately leading to glutamine-induced mTORC1 upregulation [31]. These findings imply that suppression of the mTORC1 cascade severely impacts c-MYC driven liver carcinogenesis. However, treatment approaches with Rapamycin and its analogs (Rapalogs), which are classical mTORC1 inhibitors, were proven to be ineffective in HCC clinical trials [40,41,42,43,44]. Furthermore, Rapamycin prolonged use, by inhibiting mTORC1, has been shown to paradoxically induce liver damage, inflammation, and liver tumor development, at least in some preclinical liver cancer models [45]. Thus, it is conceivable that targeting important mTORC1 downstream effectors rather than mTORC1 itself could be beneficial for the treatment of HCC. Following this hypothesis, in the present study we investigated the functional and therapeutic relevance of FASN, a mTORC1 specific target and the master regulator of de novo fatty acid biosynthesis in epithelial tissues, on hepatocarcinogenesis driven by c-MYC. We specifically selected FASN for our analysis both because of its well-established tumor supporting role in liver carcinogenesis and the fact that FASN is dispensable in the adult liver and, thus, can be suppressed without significantly harming normal cells. In addition, specific soluble inhibitors of FASN are accessible and can be applied in experimental systems [24,25,26,27,28,29,30].

As the first step of our investigation, we confirmed the elevated FASN expression in c-MYC high human HCC cell lines as well as c-Myc mouse HCC samples. Our study suggests that increased FASN expression is likely due to increased mTORC1 activation, but not directly induced by the c-MYC transcription factor. We also found that silencing of *c-MYC* gene leads to increased *FASN* mRNA expression. Intriguingly, a previous study showed that overexpression of *c-Myc* suppresses HRas induced FASN expression in a mouse model of HCC [46]. The results highlight the complicated nature of the molecular crosstalk between oncogenes and metabolic cascades in cancer. As human HCC is a highly heterogeneous disease, different oncogenic combinations may lead to distinct metabolic responses and metabolic addictions. All these data support the concepts of precision medicine for cancer treatment. 

Using in vitro growing mouse and human HCC cell lines, we found that c-MYC amply relies on FASN for its oncogenic activity. The importance of FASN on c-MYC driven growth was underscored by the strong growth restraint induced by FASN inactivation in HCC cell lines expressing high levels of c-MYC. Of note, equivalent results were obtained in human and mouse HCC cell lines, implying that the functional interplay between c-MYC and FASN is conserved among species. The observations in vitro were further substantiated by the findings in vivo. Indeed, we discovered that ablation of *Fasn* leads to a striking delay of hepatocarcinogenesis induced by co-expression of c-Myc and MCL1 in the mouse liver by hydrodynamic gene delivery. Specifically, while tumors rapidly emerged in the liver parenchyma of c-Myc/MCL1 wild-type mice, resulting in high tumor burden by 5-6 weeks post hydrodynamic injection, a comparable tumor burden was observed only 36 weeks post injection in c-Myc/MCL1 mice depleted of FASN (c-Myc/MCL1/Cre mice). An extraordinarily strong anti-neoplastic effect on hepatocarcinogenesis exerted by depletion of FASN has been previously detected by our group in HCC mouse models overexpressing the AKT protooncogene, either alone or in association with c-Met. In these models, ablation of FASN completely suppressed HCC formation, implying the need of FASN for liver tumorigenesis induced by AKT [25,30]. Similar data were reported in a recent study by Guri et al. In this study, the authors developed a mouse model consisting of loss of *Tsc1* and *Pten* tumor suppressors in the liver (L-dKO mice), leading to liver-specific overactivation of the mTOR pathway and consequent fatty acid synthesis, liver steatosis, and hepatocarcinogenesis. In these mice, both pharmacologic and genetic approaches aimed at inhibiting FASN resulted in the abolishment of HCC development [47]. Different from AKT, AKT/c-Met, and L-dKO mice, livers tumors still developed in c-Myc/MCL1/Cre mice, albeit after long latency. Thus, c-Myc/MCL1 mice possess compensatory mechanisms partly circumventing the anti-neoplastic activity imposed by FASN inactivation. In this regard, preliminary data from our group seem to exclude the relevance of the cholesterol pathway or fatty acid uptake mechanisms in the survival of tumor lesions from c-Myc/MCL1/Cre mice. However, despite the lack of a robust “compensatory” induction of the cholesterol pathway or fatty acid uptake, we found that some components of the cholesterol biosynthesis pathway, such as *Sqs* and *Lss*, as well as the fatty acid transporters *Lpl* and *Cd36*, exhibited higher expression levels in liver lesions from c-Myc/MCL1/Cre mice than in wild-type (normal) livers. Consequently, it is possible that these pathways still provide a metabolic support, although with low efficiency, to the growth of c-Myc/MCL1 liver lesions depleted of FASN. To convincingly address this important issue, specific in vivo experiments should be conducted using appropriate knockout mouse models. Although these data are preliminary and require further investigation, they are in disagreement with the finding of a partial inhibition of hepatocarcinogenesis following *Fasn* depletion in sgPten/c-Met, PIK3CA/c-Met, and PIK3CA-NRasV12 mice, a biologic event that was paralleled by the remarkable induction of the cholesterol pathway in these models [37]. Furthermore, liver tumor development was completely suppressed in sgPten/c-Met mice only when FASN ablation was coupled to inhibition of cholesterol synthesis [37]. In addition, we have shown that FASN genetic deletion did not affect liver carcinogenesis induced by overexpression of c-Met and mutant β-catenin in the mouse liver (c-Met/β-Catenin mice) [28], implying that some oncogenes and/or their combination are capable to drive the liver neoplastic process independent of FASN, and the mechanisms responsible could be multiple. 

Of note, recent reports suggest that the tumor-supporting activity of FASN might be mediated predominantly by its ability to induce genes involved in proliferation and inhibition of apoptosis, rather than by FASN lipid biosynthesis properties [30,31]. In accordance with these recent data, we found in this study that genetic depletion of Fasn resulted in the lower growth rate of c-Myc/MCL1/Cre tumors as well as in the decrease of *Cdk1*, *Cdk2*, *Skp2*, and *Survivin/Birc5* genes, which have been previously found to be regulated by FASN in cancer cells [31]. Obviously, although promising, the importance of the latter findings should be validated using appropriate in vitro and in vivo knockout models, and additional more comprehensive approaches such as transcriptomic profiling should be employed to identify the oncogenes that are affected in their malignant potential by the modulation of FASN. Furthermore, the molecular mechanisms whereby FASN positively regulates the proliferation and apoptosis of HCC cells should be defined in detail. 

The data coming from the present study have important translational implications. Our genetic studies indicate that *c-MYC*-overexpressing cells largely rely on FASN activity to exert their oncogenic properties. Several anti-FASN drugs have been generated and are current under investigation in numerous experimental models. Noticeably, TVB2640, an oral small-molecule inhibitor of FASN [48], has shown a significant activity as a single agent in multiple human solid tumors (https://clinicaltrials.gov/ct2/show/NCT02223247). These promising results were achieved with limited and reversible side effects. Furthermore, TVB2640 is currently under evaluation, alone or in combination with other drugs, in non-small cell lung carcinoma, HER2-positive advanced breast cancer, colorectal cancer, and high-grade relapsed astrocytoma as well as in subjects with non-alcoholic steatohepatitis (NCT03808558, NCT03179904, NCT02980029, NCT03032484, and NCT03938246). In addition, it has been demonstrated in experimental models that FASN inhibitors synergize with multiple chemotherapeutic agents and that FASN blockade restores the sensitivity to chemotherapeutic drugs and targeted therapies [49]. Based on these encouraging data and in the light of the problems in directly targeting the c-MYC protooncogene, the use of FASN inhibitors might represent a valid therapeutic approach for HCC, at least in the tumor subset showing activation of c-MYC. The c-Myc induced murine HCC will serve as an excellent preclinical model to evaluate the therapeutic potential of FASN inhibitor for HCC treatment, either alone or in combination with other chemotherapeutic agents. The results will provide further support for the key role of FASN and related lipogenesis-dependent and -independent mechanisms in c-MYC driven hepatocarcinogenesis. The data will also support the testing of these drugs in clinical trials.

## 4. Materials and Methods 

### 4.1. Constructs and Reagents

The constructs used in the present study, including pT3-EF1α-c-Myc [10], pT3-EF1α-MCL1, pCMV-Cre, and pCMV/sleeping beauty transposase (SB), were described previously [23]. The pT3-EF1α vector used in the current study belongs to the second-generation plasmid of this type, which lacks LoxP sites surrounding the Inverted Repeats, different from the first generation pT3-EF5α vector. Plasmids were purified using the Endotoxin-free Maxi Prep Kit (Sigma-Aldrich, St. Louis, MO, USA) before being injected into mice.

### 4.2. In Vitro Studies

Mouse liver tumor cell lines, HCC3-4, HCC4-4, which were isolated from c-Myc transgenic mice [33], and human HCC cell lines, Hep40, SNU475, Huh7, and PLC were used in this study. All cell lines, after validation (Genetica DNA Laboratories, Burlington, NC, USA) were grown in a 5% CO_2_ atmosphere, at 37 °C, in medium supplemented with 10% fetal bovine serum (FBS; Sigma-Aldrich) and penicillin/streptomycin (Sigma-Aldrich). Two FASN inhibitors, namely C75 (final concentration 50 µM; Sigma-Aldrich) [50], and FASNALL (final concentration 20 µM; Cayman Chemicals, Ann Arbor, MI, USA) [51], were administered to HCC3-4, HCC4-4, Hep40, SNU475, Huh7, and PLC cell lines for 24 h and 48 h. Cell proliferation was evaluated in the cell lines at 24- and 48-h time points using the BrdU Cell Proliferation Assay Kit (Cell Signaling Technology, Danvers, MA, USA). As concerns apoptosis, it was determined in the HCC cell lines using the Cell Death Detection Elisa plus Kit (Roche Molecular Biochemicals, Indianapolis, IN, USA), following the manufacturer’s instructions. Specifically, cells were subjected to 24 h serum starvation, and apoptosis was determined at 24- and 48-h time points. Furthermore, the partial mTORC1 inhibitor, Rapamycin (final concentration 25 µM/L, Sigma-Aldrich), was administered to HCC3-4, HCC4-4, Hep40, and SNU475 cell lines for 24 h, 44 h, 48 h, or 72 h. All cell line experiments were repeated at least three times in triplicate.

### 4.3. Knockdown of FASN, c-MYC, Raptor, and S6

For gene silencing studies, HCC3-4 and HCC4-4 mouse liver tumor cell lines were transfected with 30 pmol/L siRNA targeting mouse *Fasn* (# s65867), mouse *c-Myc* (# s70224), mouse *Raptor* (ID # 178055; Life Technologies, Carlsbad, CA, USA), or mouse *S6* (ID# 64216; Thermo Fisher Scientific, Waltham, MA, USA). Human Hep40, SNU475, Huh7, and PLC cell lines were subjected to treatment with 30 pmol/L siRNA targeting human *FASN* (# s5032), human *c-MYC* (# s9130), human Raptor (ID# s33215), or human S6 (ID# s12271; Thermo Fisher Scientific). Cell lines were transfected with Lipofectamine RNAiMAX (Thermo Fisher Scientific), according to the manufacturer’s recommendations. A scramble small interfering RNA (siRNA; # 4390846; Thermo Fisher Scientific) was used as a negative control. Experiments were repeated at least three times in triplicate.

### 4.4. Quantitative Reverse Transcription Real-Time Polymerase Chain Reaction (qRT-PCR) 

Gene Expression Assays for human *FASN* (Hs01005622_m1), *SCD* (Hs01682761_m1), *c-MYC* (Hs00153408_m1), *ACC/ACACA* (Hs01046047_m1), *ACLY* (Hs00982738_m1), *SREBF1* (Hs01088679_g1), *HMGCR* (Hs00168352_m1), *SQS/FDFT1* (Hs00926054_m1), *SREBF2* (Hs01081784_m1), *LSS* (Hs01552331_m1), *LPL* (Hs00173425_m1), CD36 (Hs00354519_m1), and *β-Actin* (4333762T), as well as for mouse *Fasn* (Mm00662319_m1), *Acc/Acaca* (Mm01304257_m1), *Acly* (Mm01302282_m1), *Scd1* (Mm00772290_m1), *Scd2* (Mm01208542_m1), *Srebf1* (Mm00550338_m1), *Cdk1* (Mm00772472_m1), *Cdk2* (Mm00443947_m1), *Skp2* (Mm00449925_m1), *Birc5/Survivin* (Mm00599749_m1), *c-Myc* (Mm00662319_m1), *Hmgcr* (Mm01282499_m1), *Mvk* (Mm00445773_m1), *Sqs/Fdft1* (Mm01598574_g1), *Srebf2* (Mm01306292_m1), *Lpl* (Mm00434764_m1), *Lss* (Mm00461312_m1), *Cd36* (Mm00432403_m1), and *β-Actin* (Mm00607939_s1) genes were purchased from Applied Biosystems (Foster City, CA, USA). Quantitative values for each gene were calculated by using the PE Biosystems Analysis software and expressed as number target (NT). NT = 2^−ΔCt^, wherein the ΔCt value of each sample was calculated by subtracting the average Ct value of the target gene from the average Ct value of the *β-Actin* gene. Experiments were repeated three times in triplicate.

### 4.5. Protein Extraction and Western Blot Analysis

Mouse livers tissues were homogenized in M-PER Mammalian Protein Extraction Reagent (Thermo Fisher Scientific) containing the Complete Protease Inhibitor Cocktail (Roche Molecular Biochemicals, Indianapolis, IN, USA). Protein concentrations were determined with the Bio-Rad Protein Assay Kit (Bio-Rad, Hercules, CA, USA) using bovine serum albumin as standard. For Western blotting, aliquots of 40 μg were denatured by boiling in Tris-MOPS-SDS Running Buffer, separated by SurePAGE (Genscript, Piscataway, NJ, USA), and transferred onto nitrocellulose membranes (Bio-Rad) by electroblotting in Towbin buffer (25 mM Tris, 195 mM glycine, and 20% methanol). Membranes were blocked in Pierce Protein-free Tween 20 Blocking Buffer (Thermo Fisher Scientific) for 1 h and probed with the following specific antibodies from Cell Signaling Technology: anti-FASN (# 3180), MCL1 (# 39224), p-S6 (# 2216, 2211, and 4858), PKM1 (# 7067), PKM2 (# 4053), HK1 (# 2024), HK2 (# 2867), p-4EBP1 (# 2855), and PCNA (# 2586). The anti-HK4 antibody (# SC-7908) was obtained from Santa Cruz Biotechnology (Santa Cruz, CA, USA). Each primary antibody was followed by incubation for 30 min with horseradish peroxidase secondary antibody (Jackson ImmunoResearch Laboratories, Inc., West Grove, PA, USA), diluted 1:5000. Anti-GAPDH (EMD Millipore, Burlington, MA, USA) and anti-β-Actin (Sigma-Aldrich) antibodies were used as loading controls and proteins were revealed with the Clarity Western ECL Substrate (Bio-Rad). Western blot analysis was repeated at least three times.

### 4.6. Hydrodynamic Tail Vein Injection and Mouse Monitoring

The *Fasn^fl/fl^* conditional knockout mouse (maintained on a C57BL/6 background) has been described previously in detail [25,36]. Hydrodynamic tail vein injection was performed as reported in detail before [52]. In brief, 10 μg pT3-EF1α-c-Myc and 10 μg pT3-EF1α-MCL1 and 40 μg pCMV or 40 μg pCMV-Cre along with sleeping beauty transposase (SB) in a ratio of 25:1 were diluted in 2 mL saline (0.9% NaCl), then filtered through 0.22 μm filter, and injected into the lateral tail vein of 6 to 8-week-old *Fasn^fl/fl^* mice in 5 to 7 s. The care and use of mice for this study were carried out with the approval of the Institutional Animal Care and Use Committee (IACUC, AN108577, 9 December 2004) of the University of California, San Francisco.

### 4.7. Histology, Immunohistochemistry, and Assessment of Proliferation and Apoptosis

Liver lesions, fixed in 4% paraformaldehyde overnight at 4 °C and embedded in paraffin, were evaluated by a board-certified pathologist and liver expert (M.E. and S.R.) in accordance with the criteria by Frith et al. [53]. For immunohistochemistry, antigen retrieval was performed in 10 mM sodium citrate buffer (pH 6.0) by placement in a microwave on high for 10 min, followed by a 20-min cool down at room temperature. After a blocking step with the 5% goat serum and Avidin-Biotin blocking kit (Vector Laboratories, Burlingame, CA, USA), the slides were incubated with primary antibodies overnight at 4 °C. Slides were then subjected to 3% hydrogen peroxide for 10 min to quench endogenous peroxidase activity and, subsequently, the biotin-conjugated secondary antibody was applied at a 1:500 dilution for 30 min at room temperature. Anti-FASN (# 3180) and anti-MCL1 (# 39224) antibodies were purchased from Cell Signaling Technology, Inc. (Danvers, MA, USA). Anti-c-Myc (# 32072) antibody was obtained from Abcam (Cambridge, United Kingdom), while the anti-Ki67 antibody was from Thermo Fisher Scientific (# MA5-14520). Reaction detection was achieved using the Vectastain ABC-Elite Peroxidase Kit (Vector Laboratories, # PK-6100) using ImmPACT DAB (Vector Laboratories, SK-4105) as the chromogen. Slides were counterstained with Mayer’s hematoxylin. Oil-Red-O (ORO) staining was performed using standard protocols. To assess proliferation, the proliferation index was determined in mouse HCC lesions and wild-type normal livers by counting Ki67 positive cells on at least 3000 tumor cells per mouse sample. Apoptosis index was assessed in the same mouse samples by counting Cleaved-Parp (dilution 1:50; Cell Signaling Technology; # 94885) positive cells on at least 3000 tumor cells per mouse.

### 4.8. Human Tissue Samples

Forty-two hepatocellular carcinomas were used for the study. Tumors were divided in HCC with shorter survival/poorer outcome (HCCP; *n* = 22) and longer survival/better outcome (HCCB; *n* = 20) survival, characterized by < 3 and > 3 years’ survival following partial liver resection, respectively. Patients’ clinicopathological features are summarized in Table 1. Liver tissues were collected at the University of Greifswald (Greifswald, Germany). Institutional Review Board approval was obtained at local Ethical Committee of the Medical University of Greifswald (approval code: BB 67/10; 3 June 2010), in compliance with the Helsinki Declaration. Written informed consent was obtained from all individuals.

### 4.9. c-MYC Transcriptional Activity

The transcriptional activity of c-MYC was investigated in protein nuclear extracts from human HCC samples (*n* = 42) using the c-MYC Transcription Factor Assay Kit (Abcam, Cambridge, United Kingdom), following the manufacturer’s protocol. Nuclear extracts were isolated from frozen specimens using the NE-PER™ Nuclear and Cytoplasmic Extraction Reagents (Thermo Fisher Scientific). Experiments were repeated three times in triplicate.

### 4.10. Statistical and Bioinformatic Analyses

GraphPad Prism, version 7.0 (GraphPad Software Inc., La Jolla, CA, USA) was used to evaluate statistical significance by Tukey-Kramer and Student’s t tests, and linear regression analysis. Values of *p* < 0.05 were considered significant. Data are expressed as mean ± SD. *C-Myc* putative transcription factor binding motifs on the human and mouse *FASN* gene promoter were predicted using the Eukaryotic Promoter Database New (EPDnew; https://epd.epfl.ch), which combines EPD promoters with promoter-specific high-throughput data [35].

## Figures and Tables

**Table 1:** Clinicopathological features of hepatocellular carcinoma (HCC) patients.

Variables	
No. of patients	42
Male	31
Female	11
Age (years)	
<60	12
>60	30
Etiology	
HBV	20
HCV	14
Ethanol	6
NA	2
Liver cirrhosis	
Yes	31
No	11
Edmondson and Steiner grade	
II	12
III	18
IV	10
Tumor size (cm)	
<3	12
>3	30
Alpha-fetoprotein secretion	
<300 ng/mL	16
>300 ng/mL	26
Prognosis	
Better (≥3 years)	20
Poorer (<3 years)	22

Abbreviations: NA, not available.

## Supplementary Materials

Supplementary materials can be found at https://www.mdpi.com/1422-0067/21/22/8467/s1.

## Abbreviations

HCC	Hepatocellular carcinoma
FASN	Fatty Acid Synthase
mTORC1	Rapamycin Complex 1
MCL1	Myeloid cell leukemia 1
PKLR	Pyruvate Kinase L/R
PKM1/2	Pyruvate kinase isozymes
BCL2	B-cell lymphoma 2
ACLY	ATP citrate lyase
ACC	Acetyl-CoA carboxylase
SCD	Stearoyl-coA desaturase
SB	Sleeping beauty transposase
p-S6	Phosphorylated/activated S6
p-4EBP1	Inactivated/phosphorylated 4EBP1
HMGCR	Hydroxy-Methylglutaryl-CoA Reductase
MVK	Mevalonate Kinase
SQS	Squalene Synthase
LSS	Lanosterol Synthase
SREBF2	Sterol Regulatory Element Binding Transcription Factor 2
LPL	Lipoprotein lipase
HK	Hexokinase
