# Supplementary material for: Pivotal Role of Fatty Acid Synthase in c-MYC Driven Hepatocarcinogenesis
Source: Int J Mol Sci. 2020 Nov 11;21(22):8467. doi: 10.3390/ijms21228467 (PMC7696085; doi:10.3390/ijms21228467)
Supplement: Supplementary file 1 [file ijms-21-08467-s001.zip › Supplementary File/Supplementary Figure 4.pdf]

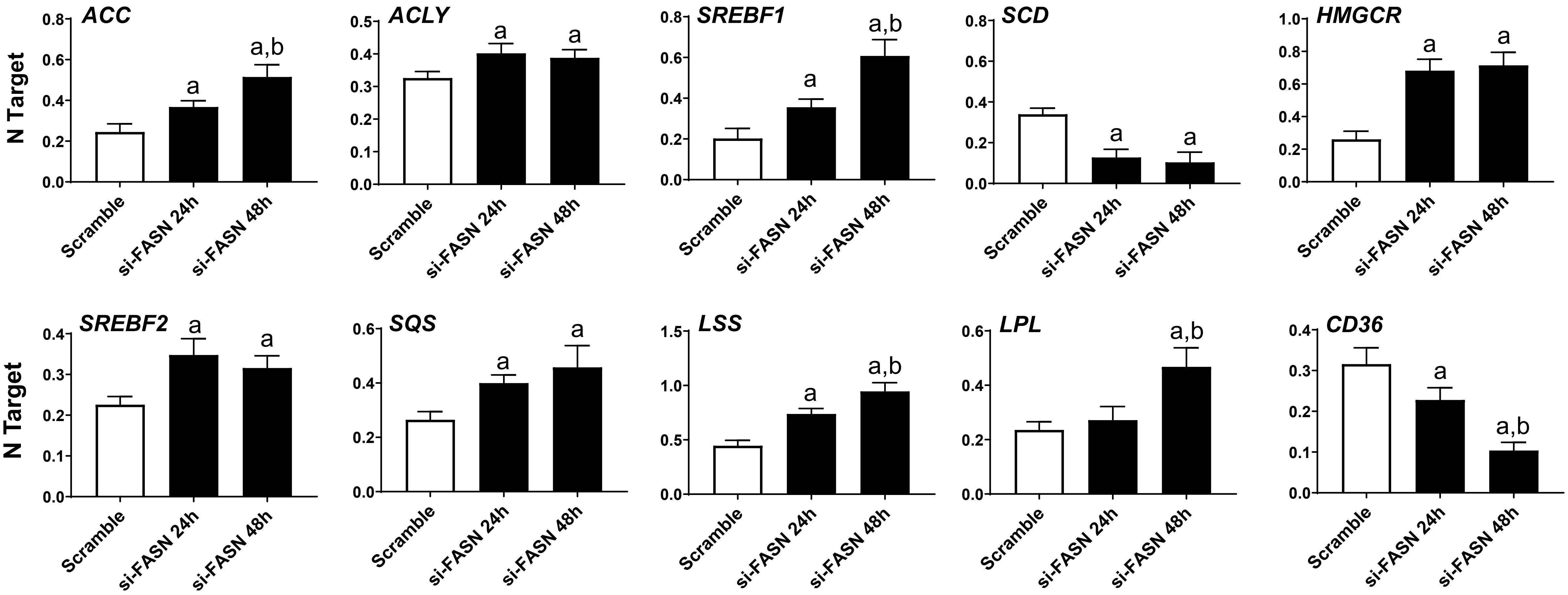

Supplementary Figure 4. Effects of suppression of FASN by FASN-specific siRNA (si-*FASN*) on the levels of major lipogenic genes in the PLC human HCC cell line, as assessed by quantitative real-time RT-PCR. Experiments were conducted three times in triplicate. Tukey–Kramer test: at least $p < 0.01$; a, vs. Scramble; b, vs. si-*FASN* 24 h.
