# Supplementary material for: Pivotal Role of Fatty Acid Synthase in c-MYC Driven Hepatocarcinogenesis
Source: Int J Mol Sci. 2020 Nov 11;21(22):8467. doi: 10.3390/ijms21228467 (PMC7696085; doi:10.3390/ijms21228467)
Supplement: Supplementary file 1 [file ijms-21-08467-s001.zip › Supplementary File/Supplementary Figure 5.pdf]

A

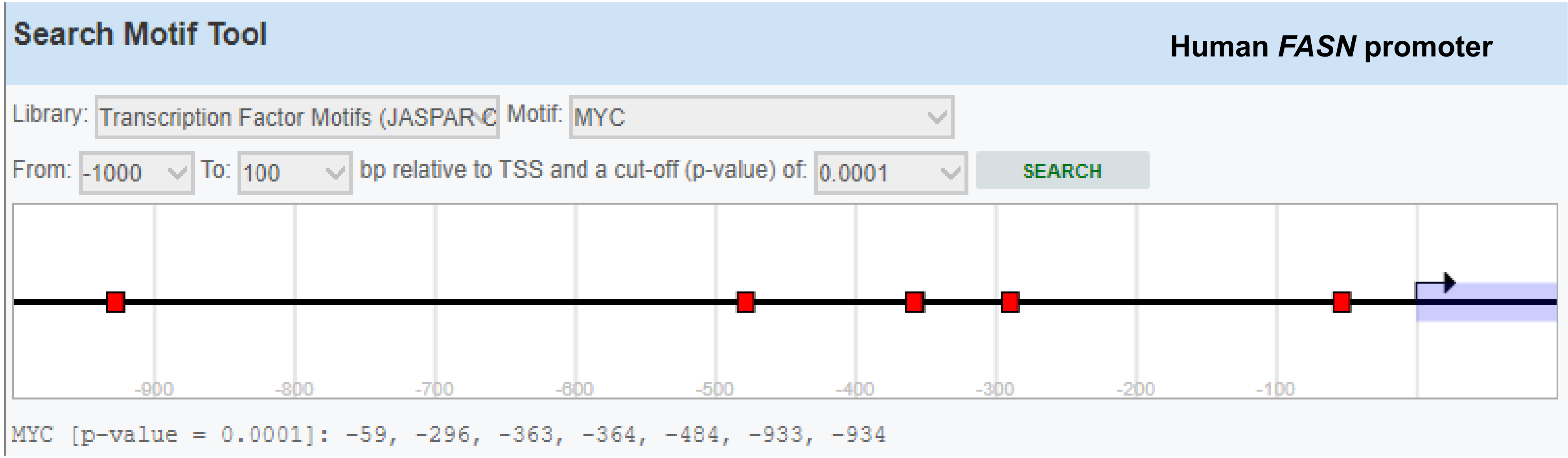

Search Motif Tool
Human FASN promoter
Library: Transcription Factor Motifs (JASPAR C
Motif: MYC
From: -1000 To: 100 bp relative to TSS and a cut-off (p-value) of: 0.0001
SEARCH
-900
-800
-700
-600
-500
-400
-300
-200
-100
MYC [p-value = 0.0001]: -59, -296, -363, -364, -484, -933, -934

B

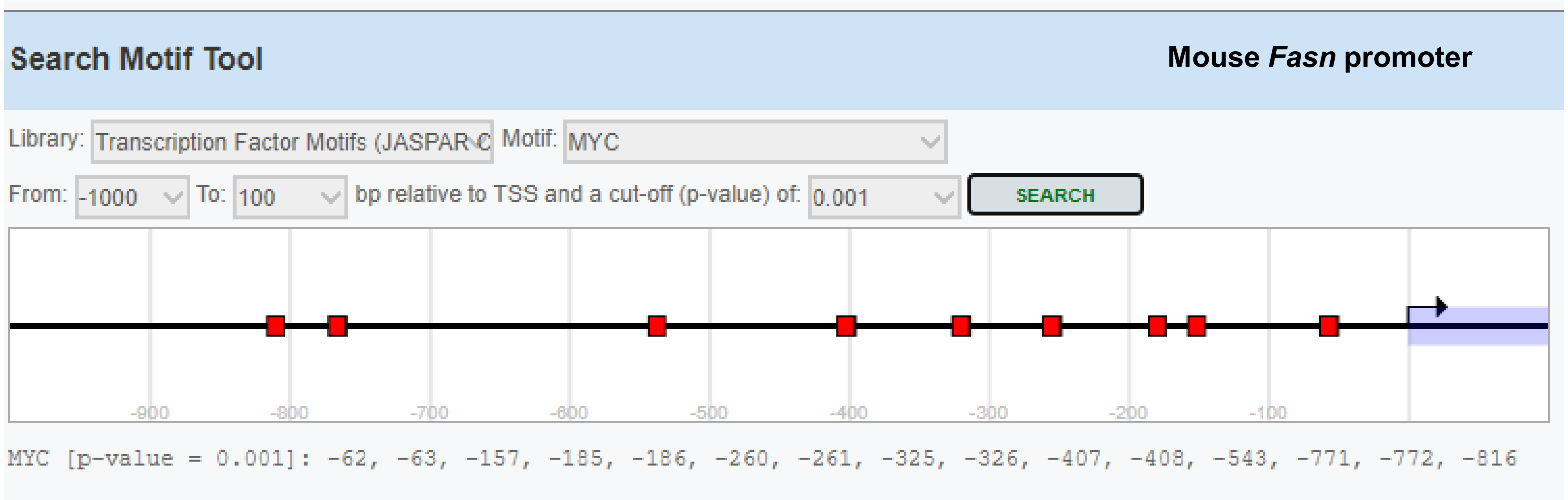

Search Motif Tool
Mouse Fasn promoter
Library: Transcription Factor Motifs (JASPAR C
Motif: MYC
From: -1000 To: 100 bp relative to TSS and a cut-off (p-value) of: 0.001
SEARCH
-900
-800
-700
-600
-500
-400
-300
-200
-100
MYC [p-value = 0.001]: -62, -63, -157, -185, -186, -260, -261, -325, -326, -407, -408, -543, -771, -772, -816

Supplementary Figure 5. In silico prediction of c-Myc binding sites on the *FASN* human and mouse gene promoter. **(A)** Putative c-Myc binding sites on the human *FASN* promoter using a cut-off *p*-value of 0.0001. **(B)** Putative c-Myc binding sites on the mouse *Fasn* promoter using a cut-off *p*-value of 0.001. Binding sites are represented in red squares. The EPDnew software[33] was used for this scope.
