# Supplementary material for: Pivotal Role of Fatty Acid Synthase in c-MYC Driven Hepatocarcinogenesis
Source: Int J Mol Sci. 2020 Nov 11;21(22):8467. doi: 10.3390/ijms21228467 (PMC7696085; doi:10.3390/ijms21228467)
Supplement: Supplementary file 1 [file ijms-21-08467-s001.zip › Supplementary File/Supplementary Figure 6.pdf]

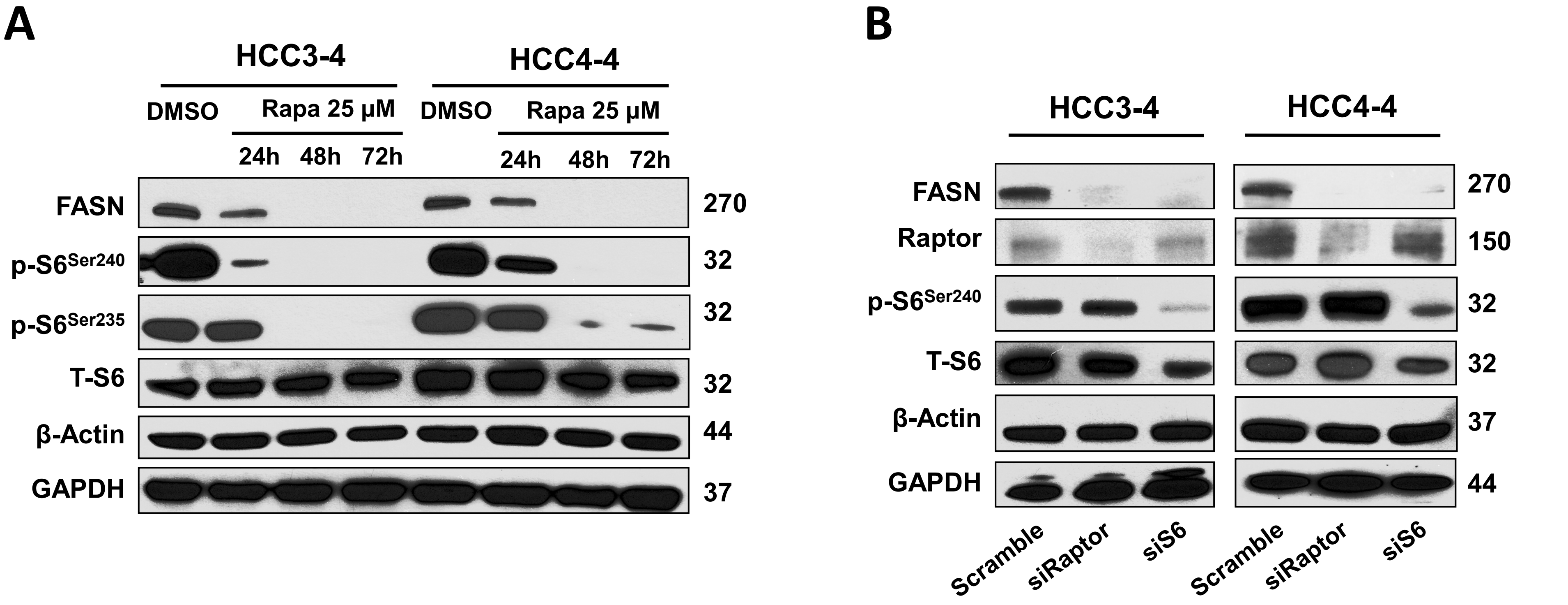

Supplementary Figure 6. FASN is regulated by the mammalian target of Rapamycin complex 1 (mTORC1) in c-Myc mouse HCC3-4 and HCC4-4 cell lines, as assessed by Western blot analysis. **(A)** Treatment with the partial mTORC1 inhibitor Rapamycin (Rapa) suppresses FASN levels in the two cell lines. As expected, levels of activated/phosphorylated ribosomal protein S6 (S6), a canonic mTORC1 target, are also downregulated in the same cells by Rapamycin. Total levels (T) of S6 remained unaffected. **(B)** Silencing of either the mTORC1 component, Raptor, or the mTORC1 downstream effector, S6, downregulates FASN in HCC3-4 and HCC4-4 cell lines. DMSO (drug solvent) was used as a control. Experiments were performed three times in triplicate. GAPDH and β-Actin protein levels were used as loading controls.
