# Supplementary material for: Pivotal Role of Fatty Acid Synthase in c-MYC Driven Hepatocarcinogenesis
Source: Int J Mol Sci. 2020 Nov 11;21(22):8467. doi: 10.3390/ijms21228467 (PMC7696085; doi:10.3390/ijms21228467)
Supplement: Supplementary file 1 [file ijms-21-08467-s001.zip › Supplementary File/Supplementary Figure 7.pdf]

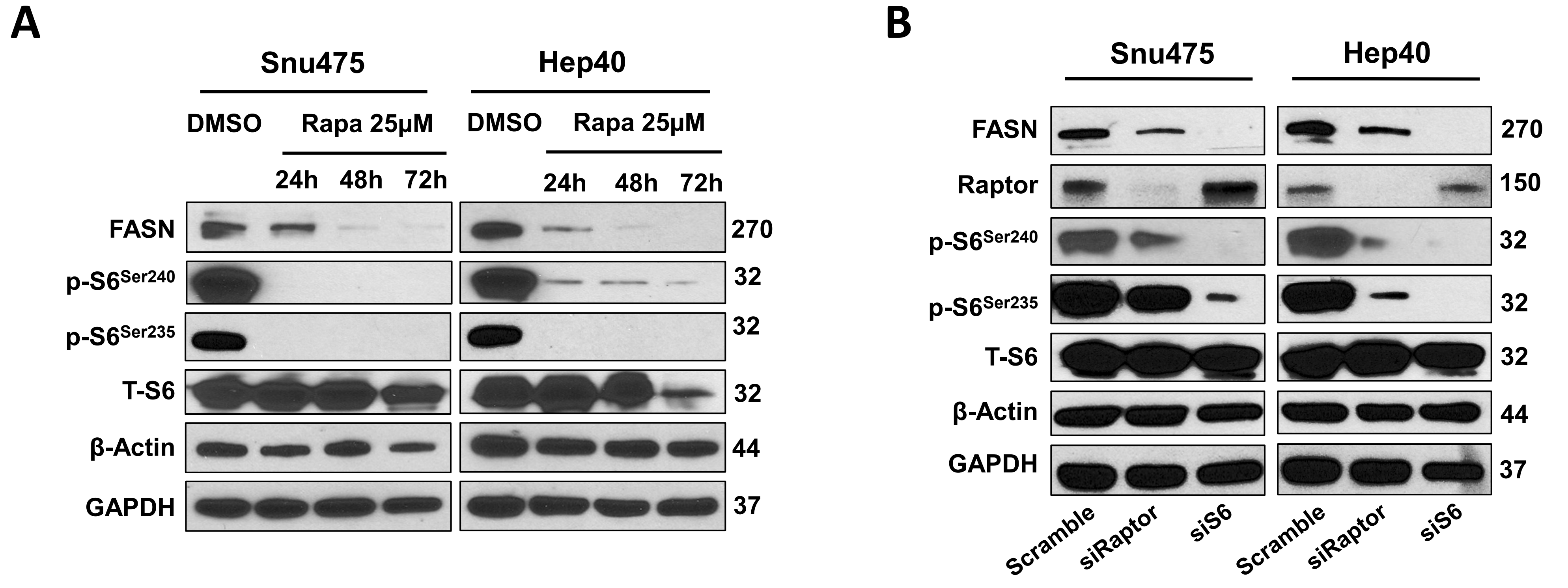

Supplementary Figure 7. FASN is regulated by the mammalian target of Rapamycin complex 1 (mTORC1) in human Snu475 and Hep40 HCC cell lines, as assessed by Western blot analysis. **(A)** Treatment with the partial mTORC1 inhibitor Rapamycin (Rapa) suppresses FASN levels in the two cell lines. As expected, levels of activated/phosphorylated ribosomal protein S6 (S6), a canonic mTORC1 target, are also downregulated in the same cells by Rapamycin. Total levels (T) of S6 remained unaffected. (B) Silencing of either the mTORC1 component, Raptor, or the mTORC1 downstream effector, S6, downregulates FASN in Snu475 and HCC4-4 cell lines. DMSO (drug vehicle) was used as a control. Experiments were performed three times in triplicate. GAPDH and β-Actin protein levels were used as loading controls.
