# Supplementary material for: Pivotal Role of Fatty Acid Synthase in c-MYC Driven Hepatocarcinogenesis
Source: Int J Mol Sci. 2020 Nov 11;21(22):8467. doi: 10.3390/ijms21228467 (PMC7696085; doi:10.3390/ijms21228467)
Supplement: Supplementary file 1 [file ijms-21-08467-s001.zip › Supplementary File/Supplementary Figure 8.pdf]

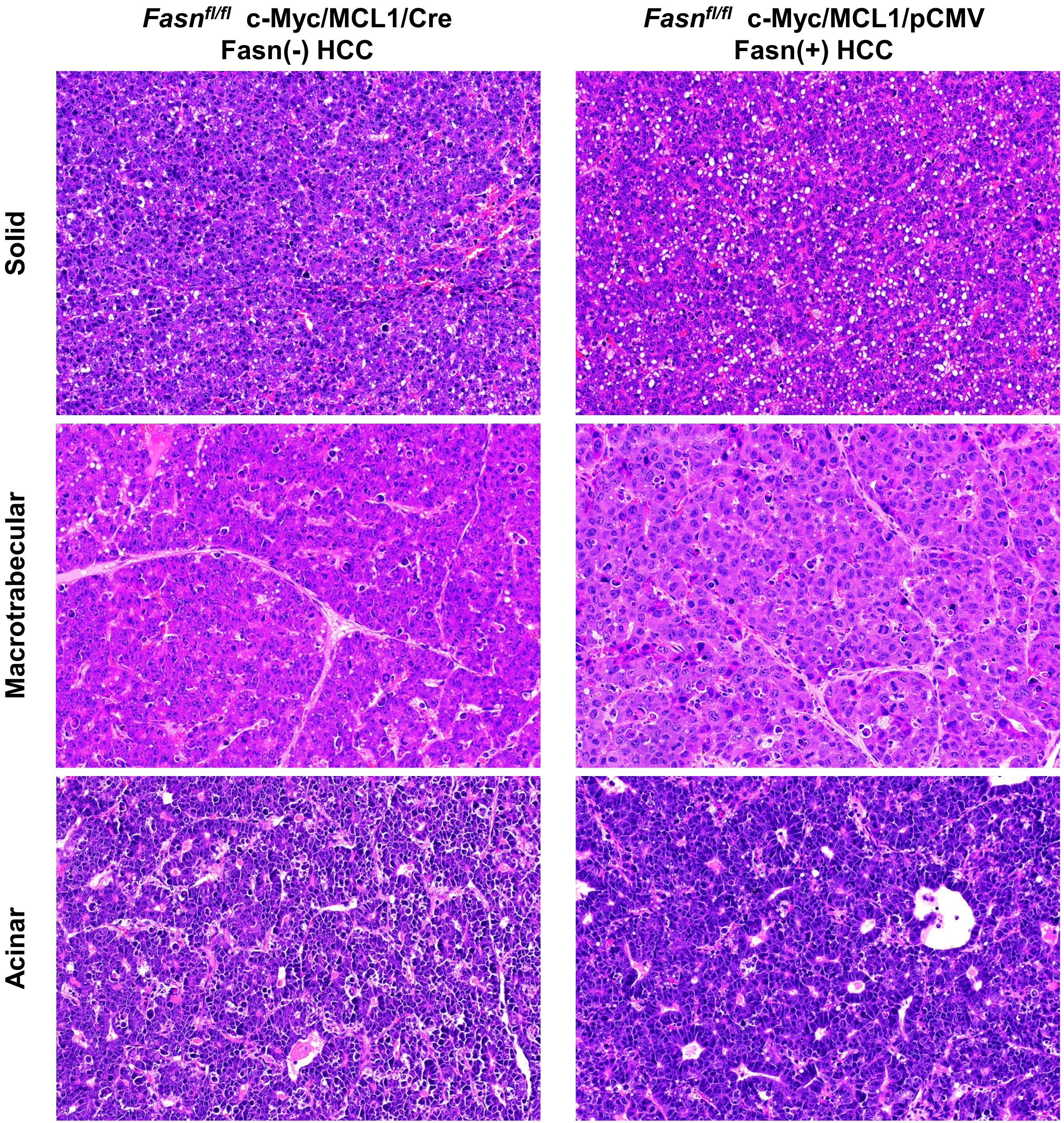

Fasn$^{fl/fl}$ c-Myc/MCL1/Cre
Fasn(-) HCC
Fasn$^{fl/fl}$ c-Myc/MCL1/pCMV
Fasn(+) HCC
Solid
Macrotrabecular
Acinar

Supplementary Figure 8. Genetic deletion of FASN does not affect the phenotype of liver tumors in c-Myc/MCL1 mice. Histopathological features of liver tumor lesions developing in c-Myc/MCL1/pCMV (*right panel*) and c-Myc/MCL1/Cre (*left panel*) mice appeared undistinguishable. Indeed, tumors from c-Myc/MCL1/pCMV and c-Myc/MCL1/Cre mice were found to be composed of small, round and highly basophilic cells with hyperchromatic nuclei, growing either as a solid pattern (*top panels*), arranged in thick chords demarcated by a thin layer of fibrovascular stroma (macrotrabecular pattern; *middle panels*), or forming acinar/pseudoglandular structures (*lower panels*). These three growth patterns most often coexisted in liver tumors from c-Myc/MCL1/pCMV and c-Myc/MCL1/Cre mice. Original magnifications: 200× in hematoxylin and eosin stained slides.
