# Supplementary material for: Pivotal Role of Fatty Acid Synthase in c-MYC Driven Hepatocarcinogenesis
Source: Int J Mol Sci. 2020 Nov 11;21(22):8467. doi: 10.3390/ijms21228467 (PMC7696085; doi:10.3390/ijms21228467)
Supplement: Supplementary file 1 [file ijms-21-08467-s001.zip › Supplementary File/Supplementary Figure 9.pdf]

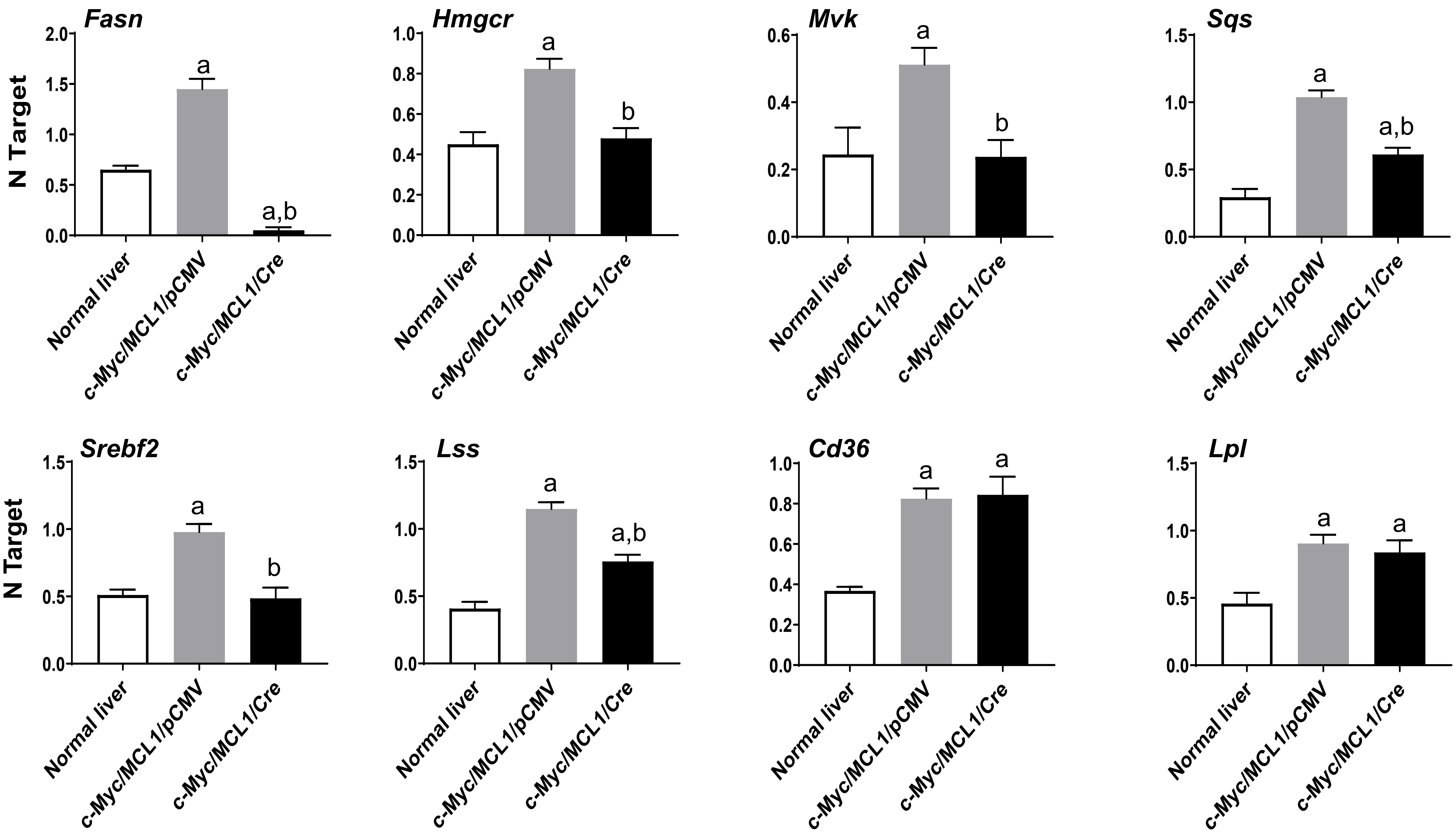
Fasn
Hmgcr
Mvk
Sqs
Srebf2
Lss
Cd36
Lpl
N Target
a
a,b
b
Normal liver
c-Myc/MCL1/pCMV
c-Myc/MCL1/Cre
0.0
0.5
1.0
1.5
2.0
0.2
0.4
0.6
0.8

Supplementary Figure 9. Quantitative real-time RT-PCR analysis of the fatty acid synthesis (*Fasn*), cholesterogenesis (*Hmgcr, Mvk, Sqs, Srebf2,* and *Lss*), and lipid uptake (*Cd36, Lpl*) in normal livers and tumor lesions of c-Myc/MCL1/pCMV and c-Myc/MCL1/Cre mice. Quantitative values for each gene were calculated by using the PE Biosystems Analysis software and expressed as number target (NT). NT = $2^{-\Delta Ct}$, wherein ΔCt value of each sample was calculated by subtracting the average Ct value of the target gene from the average Ct value of the *β-Actin* gene. Tukey–Kramer test: at least $p < 0.01$; a, vs. normal liver; b, vs. c-Myc/MCL1/pCMV mice.
